# Monolithic Metal–Semiconductor–Metal Heterostructures Enabling Next-Generation Germanium Nanodevices

**DOI:** 10.1021/acsami.1c00502

**Published:** 2021-03-08

**Authors:** Lukas Wind, Masiar Sistani, Zehao Song, Xavier Maeder, Darius Pohl, Johann Michler, Bernd Rellinghaus, Walter M. Weber, Alois Lugstein

**Affiliations:** † Institute of Solid State Electronics, Technische Universität Wien, Gußhausstraße 25-25a, Vienna 1040, Austria; ‡ Swiss Federal Laboratories for Materials Science and Technology, Laboratory for Mechanics of Materials and Nanostructures, Feuerwerkerstrasse 39, Thun 3602, Switzerland; § Dresden Center for Nanoanalysis, Technische Universität Dresden, Helmholtzstraße 18, Dresden 01069, Germany

**Keywords:** germanium, aluminum, metal-semiconductor heterostructure, solid state reaction, thermal annealing

## Abstract

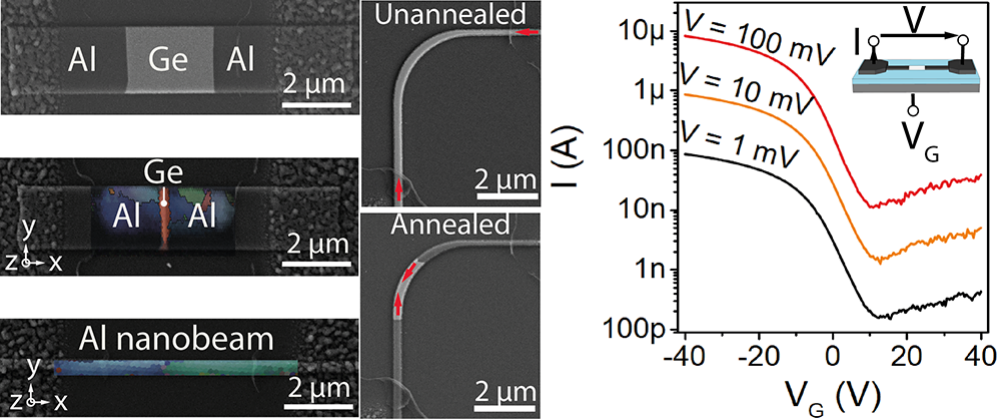

Low-dimensional Ge is perceived as a promising building block for emerging optoelectronic devices. Here, we present a wafer-scale platform technology enabling monolithic Al-Ge-Al nanostructures fabricated by a thermally induced Al-Ge exchange reaction. Transmission electron microscopy confirmed the purity and crystallinity of the formed Al segments with an abrupt interface to the remaining Ge segment. In good agreement with the theoretical value of bulk Al-Ge Schottky junctions, a barrier height of 200 ± 20 meV was determined. Photoluminescence and μ-Raman measurements proved the optical quality of the Ge channel embedded in the monolithic Al-Ge-Al heterostructure. Together with the wafer-scale accessibility, the proposed fabrication scheme may give rise to the development of key components of a broad spectrum of emerging Ge-based devices requiring monolithic metal-semiconductor–metal heterostructures with high-quality interfaces.

## Introduction

The down-scaling of integrated circuits enabled a significant reduction of the power consumption and costs of modern microelectronic devices. However, arising repercussions of short-channel effects^1^ promoted the integration of new materials, processes, and device architectures for emerging device concepts.^2,3^ In this context, low-dimensional Ge structures such as nanomembranes^4,5^ and nanowires^6,7^ have received significant attention due to their superior electrical^8,9,2^ and optical^10−12^ properties. Further, due to the ability to host superconducting pairing correlations,^13−15^ Ge gained increasing attention for encoding, processing, or transmitting quantum information.^14,16,17^ In this respect, highly transparent superconducting Al contacts^18^ are a crucial prerequisite for Josephson field-effect transistors integratable in gate-tunable superconducting qubits.^19^ Further, the combination of the s-wave superconductor Al and Ge with a strong spin-orbit coupling of holes could be an attractive candidate to study Majorana zero modes.^14,15^

Irrespective of the field of application, high-quality electrical contacts are of utmost importance and require precise nanoscale lithography. In this regard, intense research on the thermal diffusion of metals into Ge nanostructures has been carried out to achieve reliable and highly transparent germanide contacts.^20−23^ Further, material combinations with no intermetallic phase formation, such as the Al-Ge system, enabling true metal-semiconductor heterostructures with abrupt interfaces received a considerable attention.^14,24−26^

## Results and Discussion

**Figure 1:**
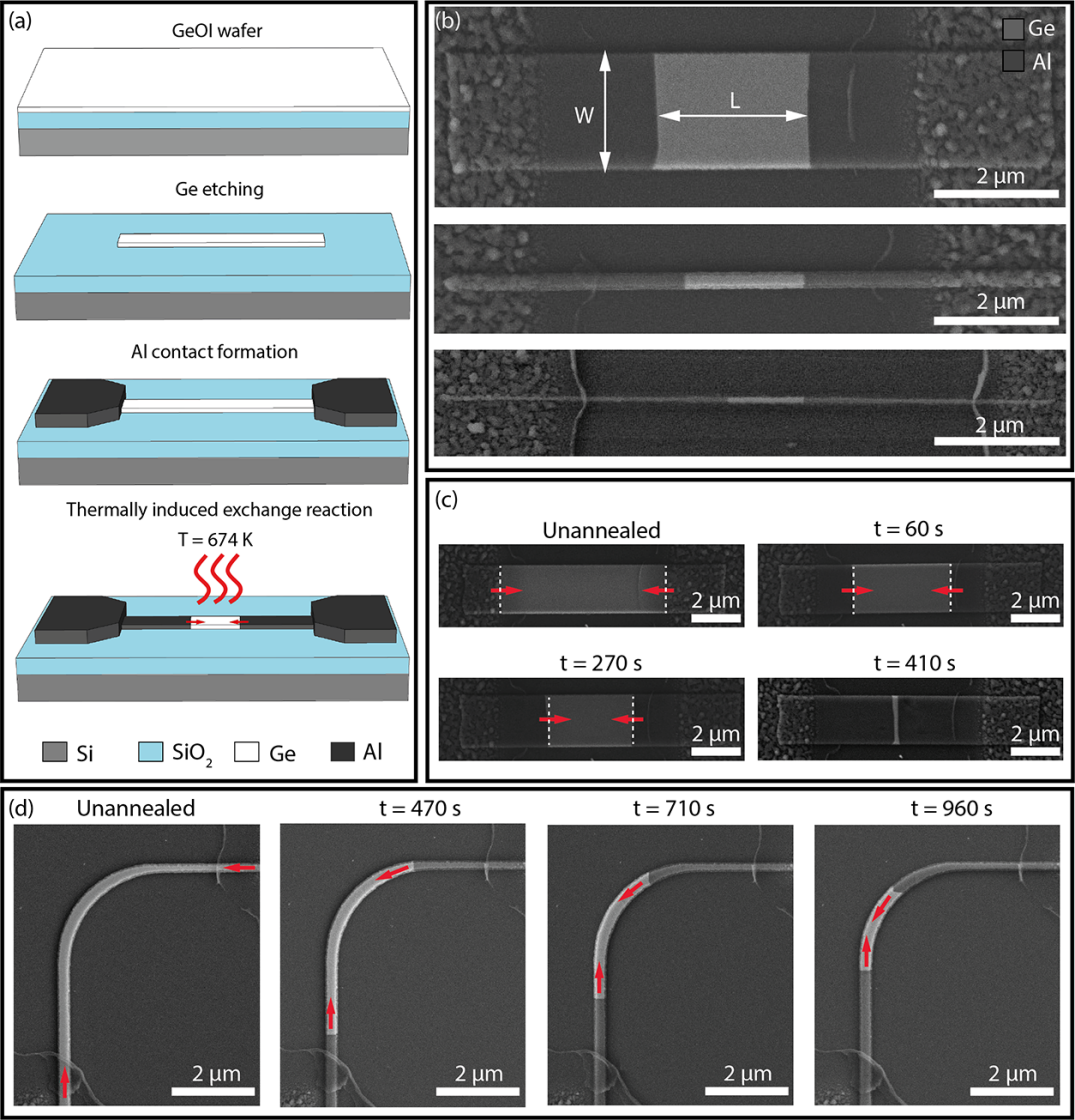
(a) Processing scheme for the wafer scale fabrication of Al-Ge-Al heterostructures, (b) SEM images of Al-Ge-Al heterostructures with different Ge channel widths (*W*) and lengths (*L*). Sequence of SEM images showing the annealing progress of a (c) straight and (d) curved Al-Ge-Al heterostructure, noting preferential faceting of the interfaces.

In this paper, we present a wafer-scale approach to achieve monolithic Al-Ge-Al heterostructures with abrupt metal-semiconductor junctions *via* a thermally induced Al-Ge exchange reaction. Without limiting the generality for parallel processing, Figure 1a shows exemplarily the fabrication scheme for an individual Al-Ge-Al heterostructure device. First, the 75 nm-thick device layer of a ⟨100⟩ oriented Ge on an insulator (GeOI) substrate is patterned using common electron beam lithography and reactive ion etching. Subsequently, Al contact pads are fabricated by optical lithography, native oxide removal, sputter deposition, and lift-off techniques (see the Methods section). Finally, the actual heterostructure formation is induced by well-controlled rapid thermal annealing in forming gas atmosphere. Figure 1b shows scanning electron microscopy (SEM) images of annealed Ge structures of different widths (*W*) brought into contact on both sides to Al pads. The images reveal dark segments emerging from the contacts, which prolong along the Ge structures during the annealing and appeared to be pure Al, embedding a monocrystalline Ge segment of length *L*.

While an Al-Ge exchange can be observed for temperatures as low as *T* = 623 K, we perform the annealing at *T* = 674 K to achieve a more controllable Al-Ge heterostructure formation. For ultrascaled Ge channels, we use consecutive annealing steps, accompanied by SEM imaging. Figure 1c shows SEM images of such an annealing sequence finally resulting in a 100 nm-long Ge channel within the monolithic Al-Ge-Al heterostructure. An SEM image of an even shorter Ge channel of just 50 nm embedded in an Al-Ge-Al heterostructure is shown in Figure S1. The thickness of the Ge channel of 75 nm is thereby determined by the thickness of the pristine device layer of the GeOI wafer. However, the formation process is not limited by the Ge thickness or geometry of the pre-patterned Ge structure. Figure 1d shows successive annealing cycles on a curved Ge nanobeam. Even more complex geometries are supplied in Figure S2. Remarkably, despite the complex surface chemistry of the Al-Ge exchange progress,^25^ all of the investigated structures reveal sharp Al-Ge interfaces even for successive annealing steps.^27^

To further show the versatility of the heterostructure formation methodology, Figure S3 shows an SEM image of a 150 nm-long Ge segment within a suspended Al-Ge-Al device fabricated by the Al-Ge exchange of an underetched Ge nanobeam.

We are convinced that the combination of a wafer-scale fabrication scheme enabling arrays of ordered Al-Ge-Al heterostructures and the versatility to fabricate complex device geometries is a significant step to advance the Al-Ge-Al heterostructure platform toward “More than Moore” architectures.

**Figure 2:**
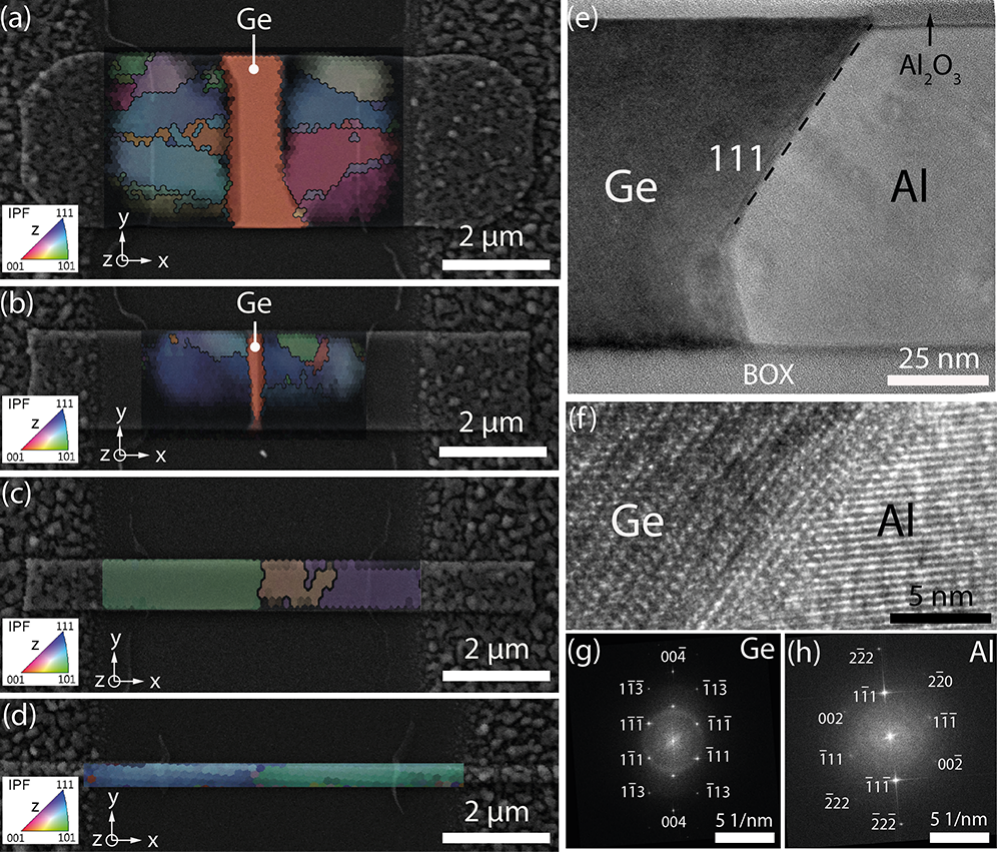
(a–d) SEM images with overlaid EBSD maps. (e) TEM and (f) HRTEM image of the Al-Ge interface revealing an abrupt Al-Ge heterojunction. The indexed FFT patterns (zone axis [110]) of the Ge and Al segments are shown in (g) and (h), respectively.

To investigate the morphology of the formed Al-Ge heterostructures in more detail, we performed electron backscatter diffraction (EBSD) and transmission electron microscopy (TEM) analysis. Figure 2a–d shows the EBSD maps of representative structures with a thickness of 75 nm and widths between *W* = 4 μm and 250 nm. For the wider structures, the formedAl contacts appeared to be polycrystalline with grain sizes in the micrometer range. For narrower structures, the number of grains decreases. Finally, for structures with *W* < 400 nm and Al contacts on both sides, long annealing time results in pure Al lines consisting of two monocrystalline segments (Figure 2d). Figure 2e shows a cross-sectional high-resolution TEM image of the Al-Ge interface. The enlarged view in the HRTEM image in Figure 2f shows a sharp Al-Ge junction. Local fast Fourier transforms (FFTs) of the Ge and Al segments are shown in Figure 2g,h, respectively. The crystal structures of the Al and Ge regions were both identified as fcc with a lattice constant *a* = 0.404975 nm and the space group 225 (ICSD 43423) in the case of Al and *a* = 0.565675 nm and the space group 227 (ICSD 43422) for Ge. Evaluating the FFTs in more detail shows that the interface is composed of a Ge {111} and an Al {200} facet, respectively. The Moirè patterns observed on the Ge side of the interface (cf. upper left corner of Figure 2f) originate from overlapping Al and Ge grains due to an inclination of the interface with respect to the electron beam direction. Both crystals are oriented in a [110] zone axis with a mutual in-plane rotation of 6.5° that may be associated with strain minimization and lattice relaxation due to the large difference in lattice constants between Al and Ge, presumably to accommodate the lattice mismatch.^28^

**Figure 3:**
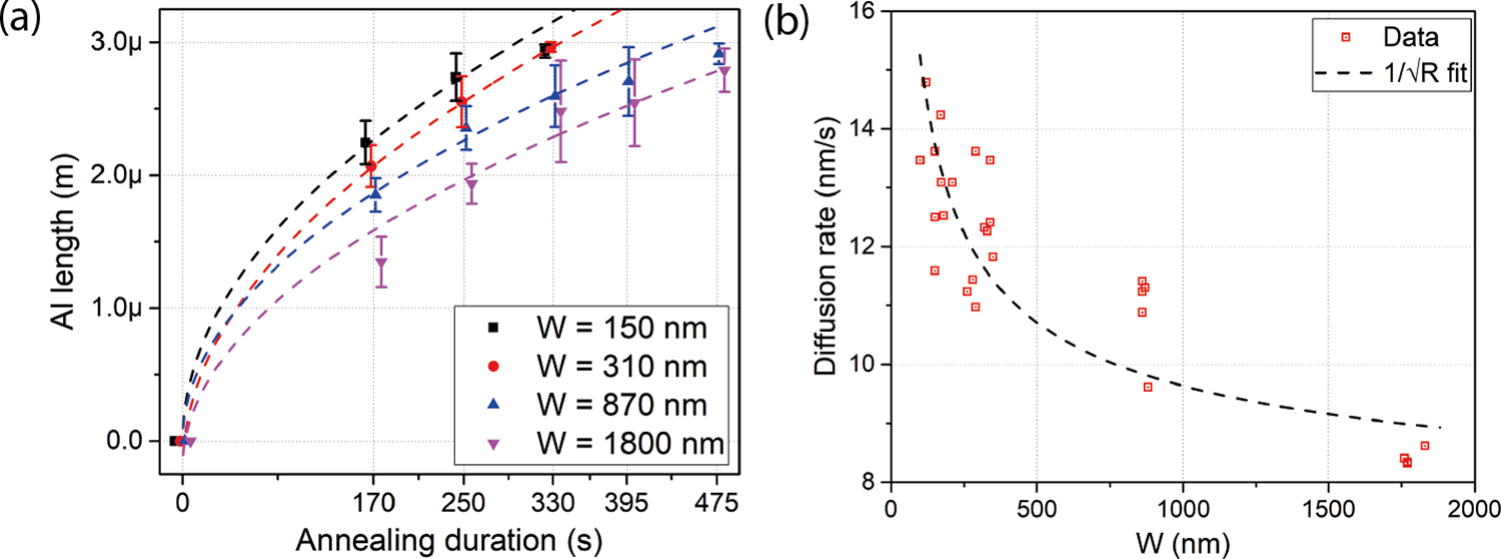
(a) Length of the formed Al contact versus annealing duration for Ge nanobeams with widths between *W* = 150 and 1800 nm and fitting curves (dashed lines) assuming a square root dependency of the Al length on the annealing duration. (b) Al-Ge exchange rate versus the width of the Ge nanobeams fitted using a 1/ function.

To investigate the kinetics of the Al-Ge exchange reaction, we monitored the progress of the Al-Ge interface during the annealing as a function of time using SEM imaging. The plot in Figure 3a shows the length of the formed Al segments as a function of the annealing duration that can be well fitted with a square root function ()*.* The overall longer Al segments for thinner structures indicate an increase of the exchange rate with decreasing structure width. The thereof calculated exchange rate together with the fitting curve visualizing the 1/ dependency of the exchange reaction is shown in Figure 3b. Based on extensive TEM investigations on the Al-Ge exchange for vapor–liquid solid^29^ grown Ge nanowires, we propose that Al propagation is governed by Ge diffusion *via* surface channels on the Al to the extended contact pads.^25^ The Al replacing the Ge is thereby provided by effective Al self-diffusion. As the Al-Ge exchange reaction rate appears to be proportional to  and inversely proportional to 1/, the diffusion of Ge from the segment to the Al contact pads can be clearly identified as the rate-limiting step.^25^ Traces of these Ge diffusion channels can be found even after the annealing as shown in the EDX mapping in Figure S4. We want to note that these Ge contaminations atop of the leads have no impact on the electrical device behavior as they are electrically short-circuited by the Al contact leads connecting the Ge channel.

To investigate the dependency of the Al-Ge exchange reaction on the crystal orientation, the Ge pattern on the (100) device layer was rotated in steps of 22.5° from the original ⟨110⟩ direction, ending up finally in a ⟨1̅10⟩-oriented Ge pattern (see Figure S5a). In accordance with similar investigations concerning the silicide formation in top-down fabricated Si nanostructures,^30^ no significant dependence of the Al-Ge exchange rate on the crystallographic orientation was found (see Figure S5b). The ability to fabricate devices with different orientations with similar Ge channel lengths provides a significant advantage with respect to the future wafer-scale integration of Al-Ge-Al heterostructures. We note that the variation in the Al-Ge exchange rate might be challenging for the fabrication of ultrascaled Ge channels. However, we are convinced that the reported Al-Ge exchange rate variation can further be optimized by process tuning with respect to annealing temperature and process time. Further, as the formation of NiSi-Si heterostructures relies on similar exchange rate variations to our system,^30^ the proposed Al-Ge system may enable “More than Moore” device concepts such as reconfigurable transistors,^31^ comprising individual source/drain overlapping gate contacts.

Notably, associated with the asymmetric diffusion coefficients in the Al-Ge material system, the diffusion of Al in Ge is extremely slow (see Table S1).^24,25,32^ Hence, in contrast to common metal-germanide alloy formation,^33^ the Al-Ge exchange results in a high-quality true metal–semiconductor heterostructure without any measureable Al contamination in the Ge segment.^25^

**Figure 4:**
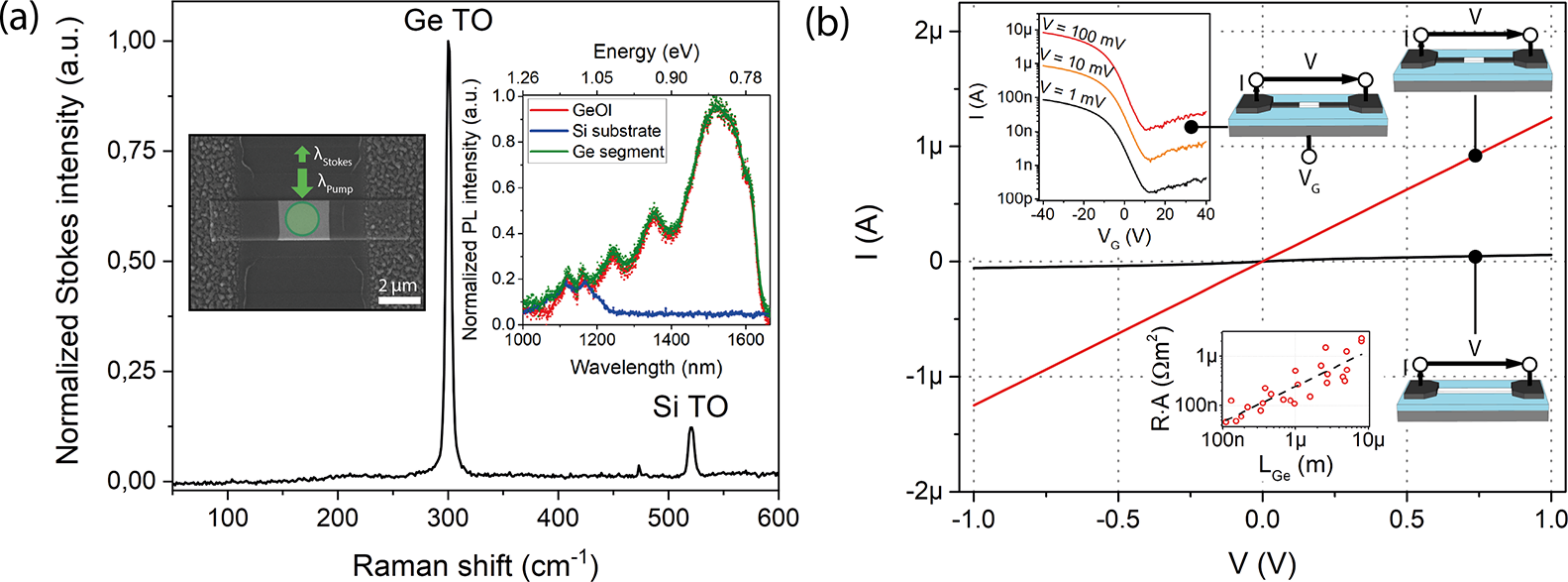
(a) *Normalized* Stokes Raman spectra of the Ge channel of an Al-Ge-Al heterostructure with a width of *W* = 2 μm and a Ge length of *L* = 1.5 μm for laser excitation (λ_Pump_ = 532 nm) with 8 kW/cm^2^. The Raman spectrum shows the TO modes at 301 and 520 cm^–1^ assigned to Ge-Ge and Si-Si vibrations of the Ge channel and the Si substrate, respectively. The left inset shows the SEM image of the investigated Al-Ge-Al heterostructure device. The right inset shows a comparison of the normalized PL spectrum of the Ge channel, the GeOI wafer, and a Si substrate for a laser excitation (λ_Pump_ = 532 nm) with 20 kW/cm^2^. (b) Comparison of *I*/*V* plots of a Ge nanobeam before (black) and after annealing (red). The lower inset shows the calculated resistance of Al-Ge-Al heterostructure devices with a structural width of *W* = 2 μm as a function of Ge segment length. The upper inset shows the transfer characteristics of the device for bias voltages of *V* = 1, 10, and 100 mV.

To check the internal stress in the Ge segment, probably as part of the manufacturing process, we performed μ-Raman measurements on a 2 μm-wide and 1.5 μm-long Ge segment shown in the inset of Figure 4a. The Raman spectrum in the main plot reveals two distinct peaks assigned to the Stokes transverse optical (TO) modes of Ge at about 301 cm^–1^ and the supporting Si handle wafer at 520 cm^–1^.^34−36^ The measured full width at half-maximum of the Ge mode (Γ = 5.1 cm^–1^) in the spectrum is only ∼1.7 cm^–1^ larger than in the bulk (Γ = 3.4 cm^–1^),^37^ which indicates a pure Ge segment of good crystal quality.^38^

To evaluate the optical and electrical properties of the monolithic Al-Ge-Al heterostructures, we performed photoluminescence (PL) and common *I*/*V* measurements. The right inset of Figure 4a shows exemplarily a comparison of the room-temperature near-infrared PL spectra obtained at the Ge segment, the bare device layer, and the Si handle wafer of the GeOI substrate. The peaks at 1.01 and 0.93 eV can be assigned to Fabry–Pérot resonances^39^ due to the layered structure of the GeOI wafer and were thus also observed for the bare GeOI substrate. To identify the substrate-related PL, a measurement on the Si handle wafer with the BOX atop was added, which revealed that the double peak structures at 1.10 and 1.09 eV arise from the doped Si substrate underneath the Al-Ge-Al heterostructure.^40^ However, comparing the Ge-related PL of the Ge segment embedded in the Al-Ge-Al heterostructure with the GeOI device layer shows no significant change, indicating that the annealing does not change the optical properties of the remaining Ge segment.

Next, the electrical transport properties of the fabricated Al-Ge-Al heterostructures were probed in a two-terminal configuration. The main plot of Figure 4b shows a comparison of the *I*/*V* characteristics of a *W* = 2 μm-wide Ge segment before (black) and after the annealing (red), reducing the Ge channel length of the device from *L* = 5.9 to 1.5 μm. The heterostructure appears to be less resistive due to the reduced channel length and improved contact properties. The lower inset shows that the calculated overall resistance of the Al-Ge-Al heterostructure device with a structural width of *W* = 2 μm is directly proportional to the Ge segment length corresponding to a resistivity of ρ = 2 Ω cm. This is consistent with the resistivity of an undoped Ge device layer^41^ and thus proves the absence of any Al doping and the high purity of the Ge segment after the Al-Ge exchange. After heterostructure formation, the resistance of the device dropped by a factor 20, which is significantly more than one would expect due to the reduction of the channel by a factor of approximately 3.9. This should be associated with the contact architecture change from an Al pad atop of the Ge structure to an atomically sharp contact at the Al-Ge interface.

The contact resistance was estimated by conducting symmetric *I*/*V* measurements for small bias voltages. Thereof, the resistance was extracted and plotted as a function of the channel length *L*. Linearly fitting the data, we extracted the contact resistance at *L* = 0 nm. Further, we want to note that although the contact area between Al and Ge is significantly reduced by the Al-Ge exchange, the high-quality interface, i.e., an abrupt junction with no residual parasitic oxide between Al and Ge, might cause the lower contact resistance.^18,42^ In this respect, comparing the data of more than 10 devices before and after annealing, the contact resistance was found to decrease by roughly a factor of 5 despite the reduced contact area. To determine the height of the Schottky barrier at the Al-Ge interface, we performed temperature-dependent *I*/*V* measurements. Assuming thermionic emission, the Schottky barrier height of the Al-Ge junction can be obtained from temperature-dependent *I*/*V* measurements (see the Supporting Information). The thereof calculated Schottky barrier height was estimated to be 200 ± 20 meV, which is in perfect agreement with the theoretical value of 200 meV for Al-Ge Schottky junctions.^43^ We want to note that due to Fermi level pinning, Al-Ge junctions are known to have unbalanced Schottky barrier heights, resulting in rather small barriers for holes and large barriers for electrons.^44^

Further, as shown in the upper inset of Figure 4b, using the Si substrate as a global back gate, the Al-Ge-Al heterostructure device can be operated as a field-effect transistor. Biasing with *V* = 1, 10, and 100 mV, the device exhibits a weak ambipolar transfer characteristic. For Ge nanostructures, surface doping^45^*via* acceptor-like traps shifts of the energy band structure throughout the Ge channel, causing the commonly observed p-type behavior.^46,47^ Exemplary for the evaluation of more than 10 heterostructure devices, the transfer characteristic reveals that by applying even ultralow voltages down to *V* = 1 mV, an *I*_ON_/*I*_OFF_ ratio of about 10^3^ can be achieved.

The performed optical and electrical measurements confirm that the structural and crystalline quality of Al-Ge-Al heterojunctions fabricated in a wafer-scale technology on GeOI substrates is excellent and can be used as a prototyping platform for emerging electronic and photonic nanodevices.

## Conclusions

In conclusion, we explored a wafer-scale approach for the formation of monolithic Al-Ge-Al heterostructures. Combining EBSD, EDX, and TEM measurements, we proved the purity and crystallinity of the pure Al segments with a very sharp interface between Ge and Al. In excellent agreement with the theoretical value of bulk Al-Ge Schottky junctions, a barrier height of 200 ± 20 meV was determined. Benchmarking the fabricated Al-Ge-Al heterostructures performing μ-Raman and PL measurements showed no internal stress in the Ge segment and revealed the intrinsic optical properties of the Ge channel embedded in the monolithic Al-Ge-Al heterostructure. Most notably, the reported systematic investigations provide a significant step toward the development of a broad spectrum of emerging Ge-based devices embedded in monolithic metal-semiconductor-metal heterostructures such as CMOS-compatible nanoelectronic and photonic devices. Further, we are convinced that due to the combination of the high-quality Al-Ge interface and the inherently strong spin-orbit coupling of Ge, our Al-Ge-Al heterostructures will receive significant attention for superconductor–semiconductor hybrid quantum systems.

## Methods

### Device Fabrication

The devices were fabricated from GeOI substrates comprising a 75 nm-thick (100)-oriented Ge device layer atop of a 150 nm buried SiO_2_ layer and a 500 μm-thick doped Si substrate. The Ge structures were patterned using electron beam lithography and SF_6_-O_2_-based reactive ion etching. Al pads making contact with the Ge nanostructures were fabricated by optical lithography, a 5 s HI dip (14%) to remove any Ge oxide, 125 nm Al sputter deposition, and lift-off techniques. The Al-Ge exchange reaction was induced by rapid thermal annealing at a temperature of *T* = 674 K in forming gas atmosphere. To achieve Ge devices with short segment lengths, consecutive thermal annealing steps were applied. After each annealing step, SEM images of the devices were recorded, and the parameters for further annealing steps were adapted accordingly.

### EBSD Measurements

To prevent drift due to charging effects, the samples have been coated with 2 nm of carbon prior to EBSD mapping. EBSD was performed in a Tescan MIRA SEM using a Digiview 5 camera from EDAX (UK), with beam conditions of 20 kV and 5 nA. A step size of 100 nm has been used for the mapping.

### TEM and EDX Measurements

Samples for HR(S)TEM measurements are prepared using a focused ion beam lift-out technique in an FEI Helios Nanolab 660. HRTEM images have been acquired at a FEI Titan3 80-300 image-corrected microscope. EDX measurements have been performed at a JEOL F200 microscope operated at 200 kV using a 100 mm^2^ window-less silicon drift detector. The EDX spectra have been denoised with principal component analyses (PCA) using 20 components.^48^

### Electrical Characterization

The electrical measurements were performed using a combination of a semiconductor analyzer (HP 4156B) and a probe station placed in a dark box. Temperature-dependent measurements were performed in a vacuum at a background pressure of approximately 5 × 10^–6^ mbar using a cryogenic probe station (LakeShore PS-100) and a semiconductor analyzer (Keysight B1500A).

### Optical Characterization

A confocal multifunctional microscope setup (Alpha300, WITec) equipped with a frequency doubled Nd:YAG laser emitting linearly polarized light at λ = 532 nm was employed for the optical characterization of the fabricated GeOI-based Al-Ge-Al heterostructures. For confocal μ-Raman measurements, a setup in backscattering geometry with a grating monochromator and a CCD camera (DV401- BV, Andor) were used. For the PL measurements in the near-infrared (NIR), a Princeton Instruments Acton SpectraPro 2300i spectrometer with a 150 g/mm grating (1.25 μm blaze wavelength), 300 mm focal length, and an Andor iDus DU491A-1.7 InGaAs detector array was used. To avoid artifacts from VIS photoluminescence in NIR spectra due to higher diffraction orders of the grating, an 800 nm long-pass filter was put into the beam path. For both the μ-Raman and the PL measurements, an achromatic Nikon EPI EPlan 100× objective (NA = 0.9, WD = 0.23 mm), enabling a diffraction limited spot size of ∼720 nm, was used.

## Supporting Information Available

SEM image of a nanobeam with a Ge channel of *L* = 50 nm, sequence of SEM images showing the annealing progress of various Al-Ge-Al heterostructure devices with different geometries, SEM image of a free-standing Al-Ge-Al nanobeam, EDX measurements showing the presence of Ge surface channels around the Al part of the heterostructure, table showing diffusion coefficients of the Al-Ge material system for *T* = 623 K, graph showing the average Al-Ge exchange rate for five orientations of the Ge nanobeams from ⟨110⟩ to ⟨1̅10⟩, and determination of the Schottky barrier height (PDF)
