# Supplementary material for: Monolithic Metal–Semiconductor–Metal Heterostructures Enabling Next-Generation Germanium Nanodevices
Source: ACS Appl Mater Interfaces. 2021 Mar 8;13(10):12393–9. doi: 10.1021/acsami.1c00502 (PMC7975277; doi:10.1021/acsami.1c00502)
Supplement: Supplementary file 1 — am1c00502_si_001.pdf [file am1c00502_si_001.pdf]

Supporting Information: Monolithic Metal-Semiconductor-Metal Heterostructures Enabling Next-Generation Germanium Nanodevices

Lukas Wind[1#], Masiar Sistani[1#], Zehao Song[1], Xavier Maeder[2], Darius Pohl[3], Johann Michler[2], Bernd Rellinghaus[3], Walter M. Weber[1], Alois Lugstein[1]*

[1] Institute of Solid State Electronics, Technische Universität Wien, Gußhausstraße 25-25a, 1040 Vienna, Austria

[2] Swiss Federal Laboratories for Materials Science and Technology, Laboratory for Mechanics of Materials and Nanostructures, Feuerwerkerstrasse 39, 3602 Thun, Switzerland

[3] Dresden Center for Nanoanalysis (DCN), Center for Advancing Electronics Dresden (cfaed), Technische Universität Dresden, Helmholtzstraße 18, 01069 Dresden, Germany

[#] These authors contributed equally

E-Mail Address of corresponding author: alois.lugstein@tuwien.ac.at

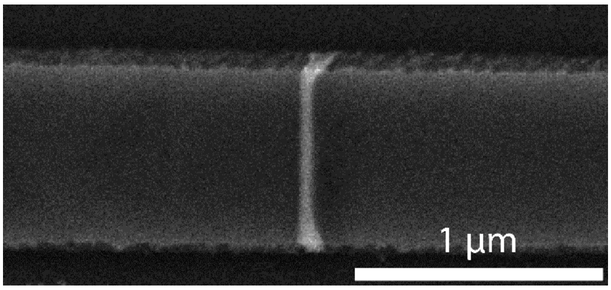

Figure S1. *SEM image of a nanobeam with a Ge channel length of L = 50 nm, showing the capability to form short Ge segments embedded in an Al-Ge-Al device architecture.*

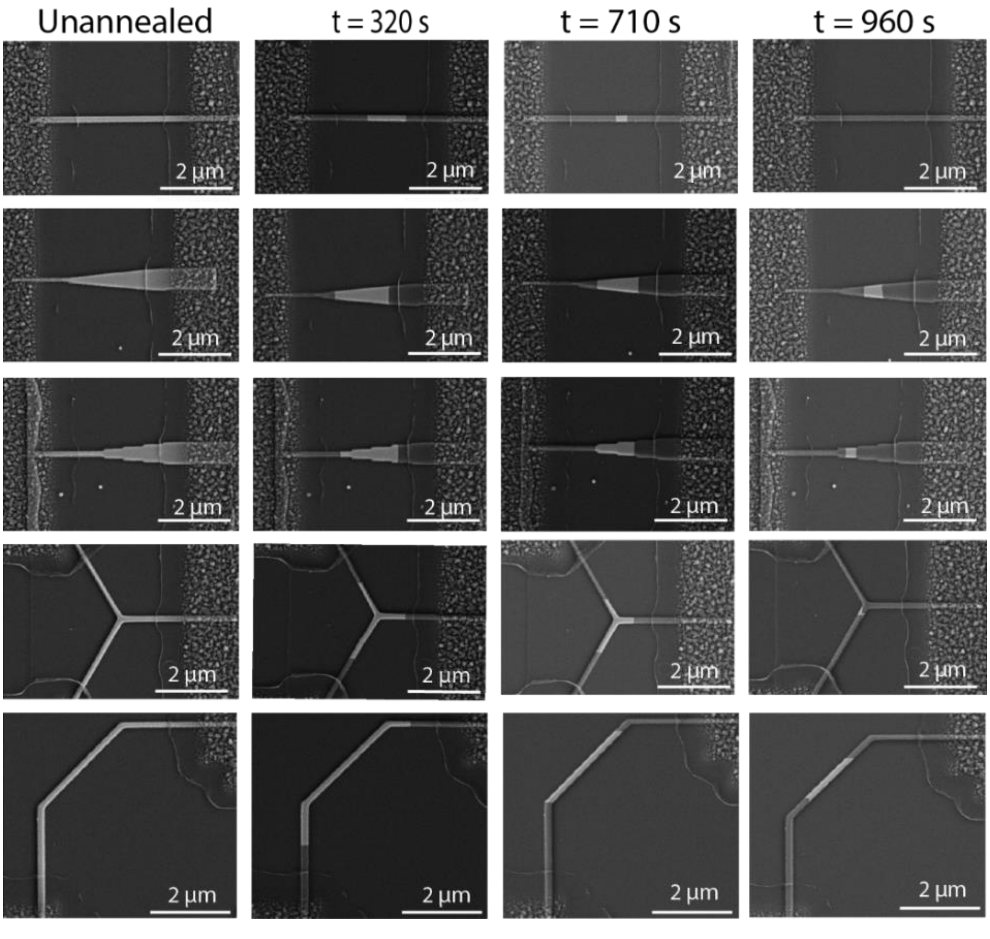

Figure S2. *Sequence of SEM images showing the formation of Al-Ge-Al heterostructure for various geometries.*

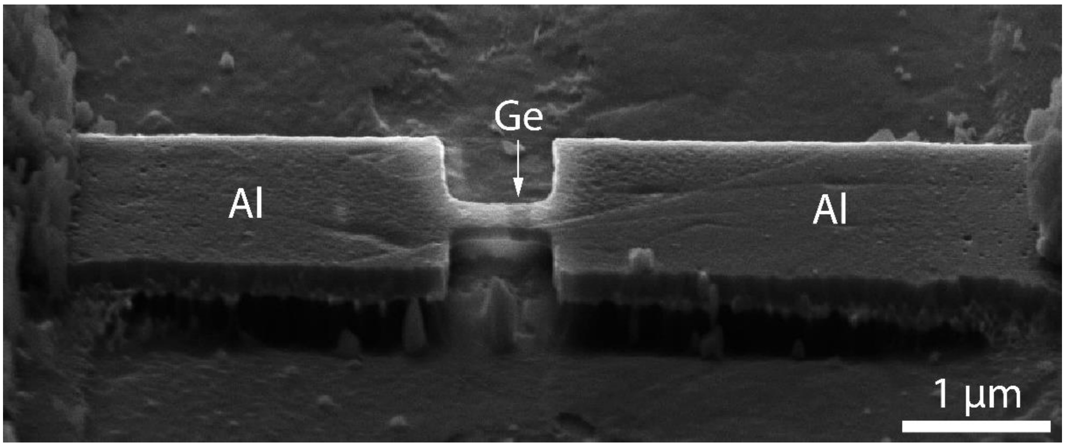

Figure S3. *SEM image showing a 150 nm long Ge channel within in a freestanding Al nanobeam.*

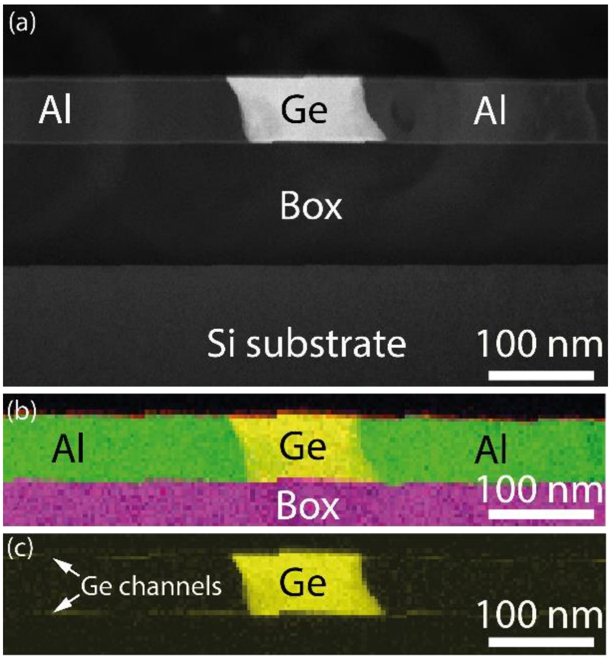

Figure S4. *(a) TEM image of the entire Al-Ge-Al heterostructure. (b) EDX measurement showing the elemental composition of the Al-Ge-Al heterostructure (Ge-yellow, Al-green, O-red, Si-purple). (c) EDX showing the presence of a Ge surface channel around the Al part of the heterostructure.*

Material	Al (cm^2s^{-1})	Ge (cm^2s^{-1})
**Al**	6.0 x 10^{-12}	1.3 x 10^{-25}
**Ge**	3.2 x 10^{-11}	9.9 x 10^{-25}

Table S1. *Diffusion coefficients of the Al-Ge material system for T = 623 K showing that the self-diffusion of Al and the diffusion of Ge in Al are 13 and 14 orders of magnitude higher compared to the self-diffusion of Ge and the diffusion of Al in Ge.*[1,2]

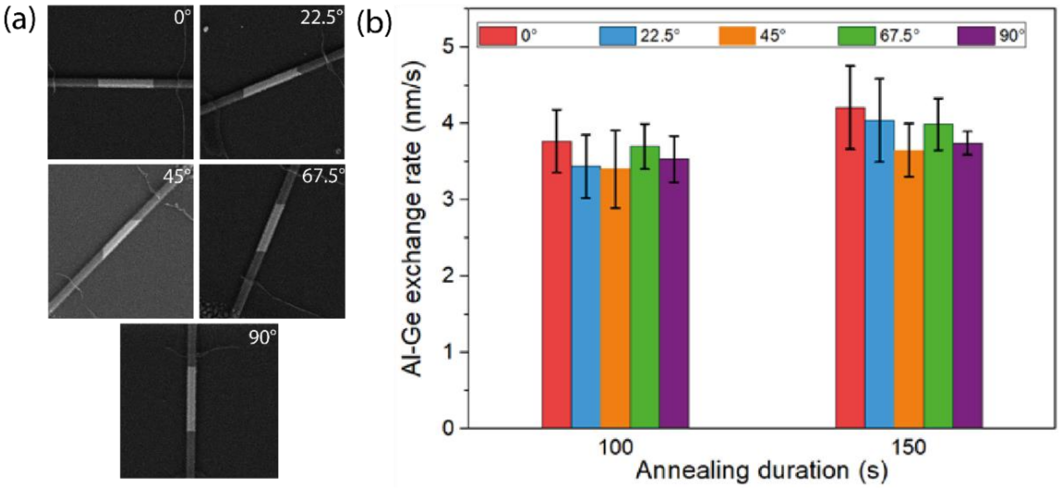

Figure S5. *(a) SEM images of Al-Ge-Al heterostructures rotated in steps of 22.5° from the ⟨110⟩ to the* $\langle\bar{1}10\rangle$ *direction. (b) Graph showing the average Al-Ge exchange rate (rectangular bars) and its scattering (error bars) for five orientations of the Ge nanobeams from* ⟨110⟩ to $\langle\bar{1}10\rangle$ *Ge for annealing durations of 100 s and 150 s at T = 674 K. The variations in the Al-Ge exchange rates (see error bars) might be associated with the quality of interface between the Al reservoirs or surface variations resulting from the subtractive nature of the top-down fabrication scheme involving reactive ion etching.*

Determination of the Schottky barrier height:

Temperature dependent I/V measurements were performed on an Al-Ge-Al heterostructure device with a structural width of W = 2 µm and a channel length of L = 4 µm to determine the Schottky barrier height of the Al-Ge junction (see Figure S5).

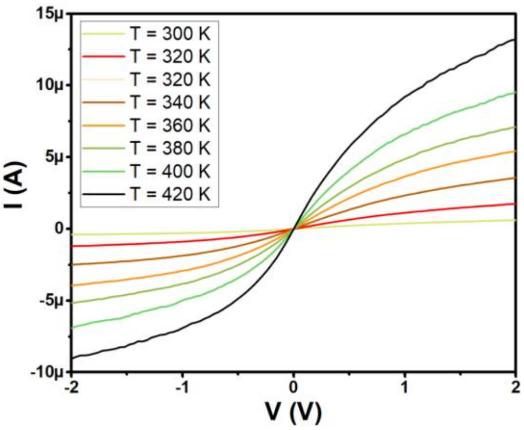

Figure S6. *I-V measurements recorded in the temperature range between T = 300 K and 420 K of an Al-Ge-Al heterostructure device with a structural width of W = 2 µm and a channel length of L = 4 µm.*

As the Al-Ge-Al heterostructure can be modeled as two back-to-back Schottky diodes, the Schottky barrier height can be extracted using the thermionic emission theory, which is valid for barrier heights much larger than $k_B T$, and small bias voltages to avoid barrier lowering and thus significant tunneling currents. The thermionic emission current over the barrier is described by $J_{TE} = A^* T^2 \exp(-\frac{q\phi_{SB}}{k_B T})$, with A^* being the effective Richardson constant. Without knowing the exact value of the Richardson constant the barrier height can be extracted by measuring the I/V characteristics at different temperatures and reformulating the previous equation to: $\ln\left(\frac{J_{TE}}{T^2}\right) = -\frac{q\phi_{SB}}{k_B T} + \ln(A^*)$. Thus, by plotting $\ln\left(\frac{J_{TE}}{T^2}\right)$ as a function of 1000/T (i.e. Richardson plot) for a specific bias voltage V the barrier height can be extracted from the slope of a linear fit, as shown in Figure S6.

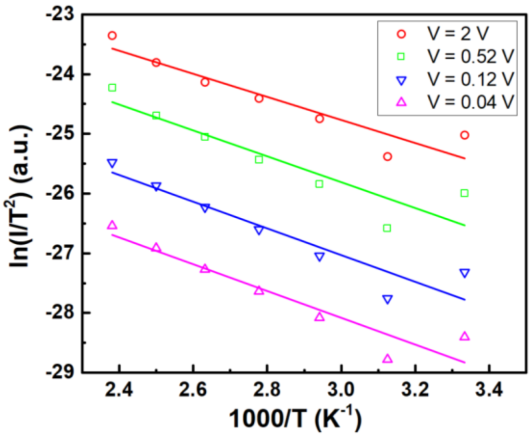

Figure S7. *Richardson plots at bias voltages ranging from 0.04 V to 2 V. The barrier height was extracted from the slope of the linear fits.*

The barrier heights $q\phi_{SB}$ are calculated from the Richardson plots and can be plotted as a function of the respective bias voltages as shown in Figure S7. Extrapolation of the data points to 0 V of several Al-Ge-Al heterostructures with similar structural sizes result in an effective barrier height of 200 +/- 20 meV, which is in good agreement with the theoretical value of 200 meV for bulk Al-Ge Schottky junctions.

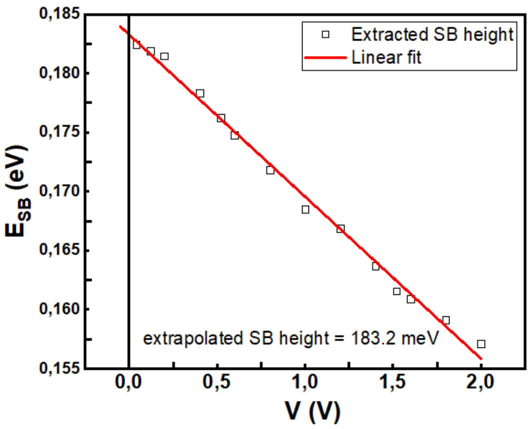

Figure S8. *Calculated Schottky barrier height as function of the applied bias. The effective Schottky barrier at 0 V bias can be extracted by extrapolation of the data points at higher bias voltages.*

REFERENCES

(1) Gale, W. F.; Totemeier, T. C. *Smithells Metals Reference Book*; Elsevier Science, 2003.

(2) Beke, D. L. *Diffusion in Semiconductors;* Landolt-Börnstein - Group III Condensed Matter; Springer-Verlag: Berlin/Heidelberg, 1998; Vol. 33A.
